# Recombinant adiponectin protects the newborn rat lung from lipopolysaccharide‐induced inflammatory injury

**DOI:** 10.14814/phy2.14553

**Published:** 2020-09-05

**Authors:** Julijana Ivanovska, Na‐Young Cindy Kang, Nikola Ivanovski, Avita Nagy, Jaques Belik, Estelle B. Gauda

**Affiliations:** ^1^ The Hospital for Sick Children Division of Neonatology, Department of Pediatrics and Translational Medicine Program University of Toronto Toronto ON Canada; ^2^ Department of Pediatric Laboratory Medicine University of Toronto Toronto ON Canada

**Keywords:** adiponectin, adiponectin receptors 1 and 2, bronchopulmonary dysplasia, chemokines, cytokines, neonatal rat

## Abstract

Preterm infants are at high risk for developing bronchopulmonary dysplasia and pulmonary hypertension from inflammatory lung injury. In adult models, adiponectin (APN)—an adipocyte‐derived hormone—protects the lung from inflammatory injury and pulmonary vascular remodeling. Cord blood APN levels in premature infants born < 26 weeks gestation are 5% of the level in infants born at term. We previously reported the expression profile of APN and its receptors in neonatal rat lung homogenates during the first 3 weeks of postnatal development. Here, we characterize the expression profile of APN and its receptors in specific lung cells and the effects of exogenous recombinant APN (rAPN) on lipopolysaccharide‐(LPS)‐induced cytokine and chemokine production in total lung homogenates and specific lung cells. In vitro, rAPN added to primary cultures of pulmonary artery smooth muscle cells attenuated the expression of LPS‐induced pro‐inflammatory cytokines while increasing the expression of anti‐inflammatory cytokines. In vivo, intraperitoneal rAPN (2 mg/kg), given 4 hr prior to intrapharyngeal administration of LPS (5 mg/kg) to newborn rats at postnatal day 4, significantly reduced gene and protein expression of the pro‐inflammatory cytokine IL‐1ß and reduced protein expression of the chemokines monocyte chemoattractant protein (MCP‐1) and macrophage inflammatory protein‐1 alpha (MIP‐1α) in the lung. LPS‐induced histopathological changes in the lung were also decreased. Moreover, rAPN given 20 hr after intrapharyngeal LPS had a similar effect on lung inflammation. These findings suggest a role for APN in protecting the lung from inflammation during early stages of lung development.

## Graphical abstract

Extremely low birth weight infants are at increased risk of developing acute and chronic lung disease from inflammation; they have low fat stores at birth and thus low circulating levels of the adipokine, adiponectin. Adiponectin is a potent anti‐inflammatory and anti‐oxidant adipokine. Here we show that recombinant adiponectin (rAPN) given to newborn rats in the saccular stage of lung development decreases LPS‐induced expression of proinflammatory cytokines and chemokines in lung homogenates and histopathological changes in the lung as shown in this graphical abstract. LPS was given intrapharyngeally and rAPN was given intraperitoneally. rAPN was effective in decreasing lung inflammation whether it was given as prevention or rescue therapy. Safe strategies that elevate endogenous APN may be benefical to extremely premature infants at risk of lung disease.

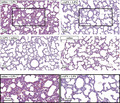

## 1 INTRODUCTION

Adiponectin (APN), a glycoprotein with several circulating isoforms, is the most abundant plasma protein and is mainly produced by adipocytes. Initially defined by its effect on metabolism, APN is a pleiotropic hormone that affects every organ system (Gauda & Master, 2018; Ruan & Dong, 2016). It has anti‐inflammatory, antioxidant, antiangiogenic, and antiatherogenic effects (Lihn, Pedersen, & Richelsen, 2005; Liu et al., 2015) and modulates cellular proliferation, autophagy, and apoptosis, thereby affecting cell growth and remodeling (Wang et al., 2005).

APN binds to AdipoR1 and AdipoR2, which are widely distributed, including in resident lung cells. It also binds to T‐cadherin, a receptor highly expressed in the vasculature that protects it from atherosclerotic injury (Fujishima et al., 2017; Matsuda et al., 2015). Binding of APN to a calreticulin (calregulin) receptor on macrophages and at recognition motifs of apoptotic cells promotes the removal of apoptotic debris by phagocytic cells and attenuates inflammation (Ouchi & Walsh, 2008; Takemura et al., 2007). All four receptors are expressed in the lung and previous in vitro and in vivo experiments support the role of APN binding to these receptors in anti‐inflammatory and anti‐oxidative protection of lung parenchyma and vasculature (Summer et al., 2008; Yamauchi, Iwabu, Okada‐Iwabu, & Kadowaki, 2014; Zana‐Taieb et al., 2015). Pulmonary APN increases nitric oxide bioavailability, prevents endothelial leak, reduces vascular smooth muscle cell proliferation and vascular remodeling (Goldstein, Scalia, & Ma, 2009; Rehan & Torday, 2012; Zana‐Taieb et al., 2015), and may have an anti‐inflammatory role by reducing the secretion of inflammatory mediators by pulmonary artery smooth muscle cells (PASMCs). Both AdipoR1 and AdipoR2 are expressed on human macrophages (Chinetti, Zawadski, Fruchart, & Staels, 2004) promoting an M2 phenotype when activated (Lovren et al., 2010), while inhibiting NF‐κB transcription and the release of inflammatory cytokines. These factors make APN an integral hormone for protecting the lung from inflammatory and oxidative injury (Chen, Yu, Xiong, Du, & Zhu, 2017; Ouchi et al., 2000).

In premature infants born during the canalicular and saccular stages of lung development, inflammation (pre‐ or postnatal) and oxidative stress increase the risk of developing bronchopulmonary dysplasia (BPD) (Hilgendorff & O'Reilly, 2015). Cord blood APN levels increase 20‐fold from 24 to 40 weeks’ gestation in human infants (Kajantie, Hytinantti, Hovi, & Andersson, 2004). Premature infants at birth have circulating APN levels of 2.2 µg/ml (0.13–12.4; [median and range]); premature infants who are intrauterine growth‐restricted and born at the limit of viability (23–24 weeks) have the lowest levels (Hansen‐Pupp et al., 2015). Thus, the low levels of APN in premature infants at birth, while sufficient for normal fetal homeostasis, may be insufficient to protect from prenatal and postnatal inflammation.

We recently published the expression profile of lung APN, AdipoR1, and AdipoR2 in rats from fetal day 19 (FD19) to postnatal day 21 (PND21) (Kang, Ivanovska, Tamir‐Hostovsky, Belik, & Gauda, 2018). In the current newborn rat study, the objectives were to describe the mRNA and protein expression profile of APN, AdipoR1, and AdipoR2 in lung cells and to characterize (a) the in vitro effect of rAPN on PASMC LPS‐induced cytokine expression and (b) the cytokine and chemokine expression in lung homogenates, expression of immune cells in air spaces, and lung histology after aspiration of LPS in PND4 rats. Our hypothesis is that exogenous APN protects the lungs from LPS‐induced injury and inflammatory influx during the saccular stage of lung development.

## 2 MATERIALS AND METHODS

Materials and methods are provided in detail in the online supplement.

### 2.1 Animal experiments

All animal procedures were approved by the Animal Care Committee of the Hospital for Sick Children and performed in accordance with the Canadian Council on Animal Care guidelines. Sprague‐Dawley rat pups on PND4 and PND7 were euthanized with a lethal dose of sodium pentobarbital, lungs were extracted and processed for primary cell cultures or flash‐frozen for protein and mRNA expression. Equal numbers of male and female animals were represented in each group. For histology, lungs were inflated, paraformaldehyde perfused, and paraffin embedded.

### 2.2 qPCR

Relative mRNA expression was determined using previously reported methods of RNA extraction, cDNA synthesis, and qPCR (Ivanovska et al., 2017). Primer sequences are listed in Table S1. Relative mRNA expression was determined by the 2∆Ct method.

### 2.3 Western blot analyses

SDS‐PAGE western blot (WB) method was used as previously described (Ivanovska et al., 2017). Antibodies, sources, and dilutions are provided in Table S2.

### 2.4 Immunofluorescent double staining of the PASMCs

Using immunofluorescence, PASMCs were double‐stained for APN, AdipoR1, or AdipoR2 (rabbit), separately paired with marker of PASMCs, α‐actin (mouse); red (Alexa Fluor 594) anti‐rabbit, and green (Alexa Fluor 488) anti‐mouse secondary antibodies were used. Images were captured using a Leica DMi8 Quorum spinning‐disc confocal microscope. Antibodies, sources, and dilution factors are listed in Table S2.

### 2.5 In vitro experiments

PND4 and PND7 lungs and pulmonary arteries were used. Explant culture technique (Ivanovska et al., 2017) for PASMC primary culture establishment was implemented. Primary cell cultures of lung epithelial cells (a mixture of airway, parenchymal, and alveolar epithelial cells) and fibroblasts were generated from whole lungs as described in Caniggia et al. (1991). All cell phenotypes were confirmed by WB detection of cell‐specific markers. Another set of PASMCs was treated with rAPN 20 µg/ml and/or LPS 3 µg/ml medium and incubated for 48 hr prior to WB analysis for cytokines (Shibata et al., 2005; Tamura, Cecon, Monteiro, Silva, & Markus, 2009).

### 2.6 In vivo experiments

#### 2.6.1 Prevention experiments

To determine whether rAPN could prevent LPS‐induced lung injury, PND4 rats were treated intraperitoneally (IP) with rAPN (2 mg/kg) or saline (Wang et al., 2006). After 4 hr (when rAPN lung concentrations peaked), intrapharyngeal administration (IPh) of LPS (5 mg/kg) or saline was administered with a pipette into the posterior pharynx (Konter et al., 2012; McGrath‐Morrow et al., 2015). After 24 hr, the pups were euthanized, with a lethal dose of pentobarbital, and lungs flash‐frozen for protein and mRNA detection. For histology, a cohort of rats was treated identically, with an additional dose of rAPN (2 mg/kg) or saline injection the following day. These pups were given a lethal dose of pentobarbital 72 hr following LPS exposure. The lungs were inflated, perfused with paraformaldehyde, paraffin embedded, sectioned and processed for histology using hematoxylin and eosin (H&E) staining. Please refer to the online supplement for a more detailed description of the inflation and fixation of the lung.

#### 2.6.2 rAPN given 20 hr after LPS (Rescue experiments)

To determine whether rAPN could rescue the lung from LPS‐induced injury, PND4 rats were given IPh LPS (5 mg/kg) or saline as outlined above. After 20 hr, the pups were given IP rAPN (2 mg/kg) or saline. Six hours after rAPN, and 26 hr after the initial LPS exposure, the pups were euthanized, and the lungs were removed and flash‐frozen and processed for protein and mRNA detection. For histology, a cohort of rats was treated identically but were given an additional dose of rAPN (2 mg/kg) 24 hr after the first rAPN dose; 72 hr after the LPS exposure, and 24 hr after the last dose of rAPN, the animals were anesthetized and the lungs were inflated, perfused with paraformaldehyde, paraffin embedded, sectioned, and processed for histology using H&E

#### 2.6.3 Lung histopathology score

A semiquantitative histopathological score was developed and used to analyze lung sections (5–6 μm) stained with H&E for the signs of inflammation focused on the presence of neutrophils and collapse of alveolar spaces when viewed at high‐power field (HPF) at 400×. Score: (a) neutrophils in the alveolar interstitium/septa (0, absent; 1 < 10 HPF; 2, 10–50 HPF; and 3, >50 per HPF); (b) neutrophils in any distal air spaces (0, absent, 1 < 5 spaces per section, 2 > 5 spaces per section; (c) neutrophils in the respiratory epithelium (lining terminal or respiratory bronchiole (0 absent, 1 present); (d) collapse of alveolar spaces (0 absent, 1 mild (one focal area per section; 2, moderate (more than one area per section); 3, extensive (over 50% of section). A total histopathology score was the sum of the individual scores and determined by a pathologist who was blinded to the treatment groups. Lung sections (three or four) were obtained from three or four animals per treatment group and used in the analysis.

### 2.7 Data presentation and analysis

Statistical analysis was performed using Sigma Plot 11.0 (Systat software, San Jose, CA, USA). All values are expressed as mean ± *SEM*, unless otherwise stated. Student's *t*‐test, and one‐way ANOVA followed by Holm–Sidak or Tukey post hoc test were used to identify significant differences between groups. A *p*‐value < .05 was considered statistically significant.

## 3 RESULTS

### 3.1 The effect of development on APN, AdipoR1, and AdipoR2 protein expression in pulmonary fibroblasts, pulmonary epithelial cells, and PASMCs

**FIGURE 1:**
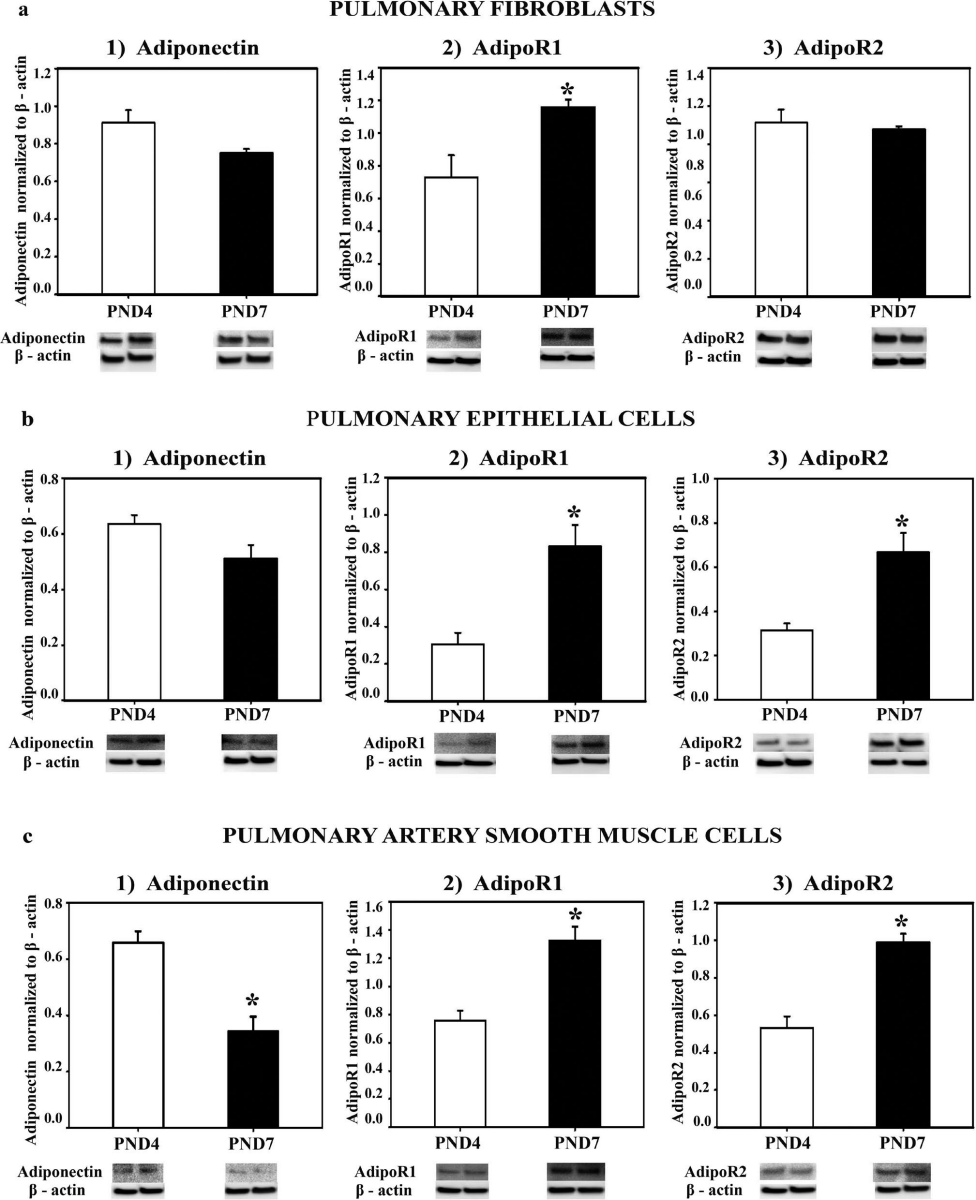
APN, AdipoR1, and AdipoR2 protein expression in lung cells in vitro. Fibroblast (a1–3), epithelial cells (b1–3), and pulmonary artery smooth muscle cells (c1–3) were isolated from lungs of Sprague‐Dawley rats at PND4 and PND7, grown in culture, homogenized, and assayed for protein expression with western blot (WB). The specificity of each cell type was preliminarily determined by WB detection of H‐caldesmon for PASMCs, EpCAM for epithelial cells, and pro‐collagen for fibroblasts (data not shown). APN, AdipoR1, AdipoR2 protein was normalized to β‐actin. Representative immunoblots are under each graph. Bars represent mean ± *SEM*. *p < .05 by *t*‐test compared to PND4. All cell experiments were done in triplicate

The saccular stage of rat lung development ends at PND4, and by PND7–8 alveolarization increases threefold (Meyrick & Reid, 1982). Using these two time periods, we determined the pattern of expression of APN and its receptors in lung cells. In all cell types examined (lung fibroblasts, lung epithelial cells, and PASMCs) AdipoR1 expression increased 1.5–2.0‐fold from PND4 to PND7 (Figure 1a2, b2, c2). While AdipoR2 protein expression did not change in lung fibroblasts (Figure 1A3) between PND4 and PND7, it increased in lung epithelial cells (Figure 1b3) and PASMCs (Figure 1c3) (*p* < .05; PND4 vs. PND7). APN expression in PASMCs decreased 1.5‐fold from PND4 to PND7 (Figure 1c1 *p* < .05); no significant decrease was seen in fibroblasts and epithelial cells (Figure 1a1, b1).

### 3.2 The in vitro effect of rAPN on PASMC cytokine production in response to LPS, at PND4 and PND7

**FIGURE 2:**
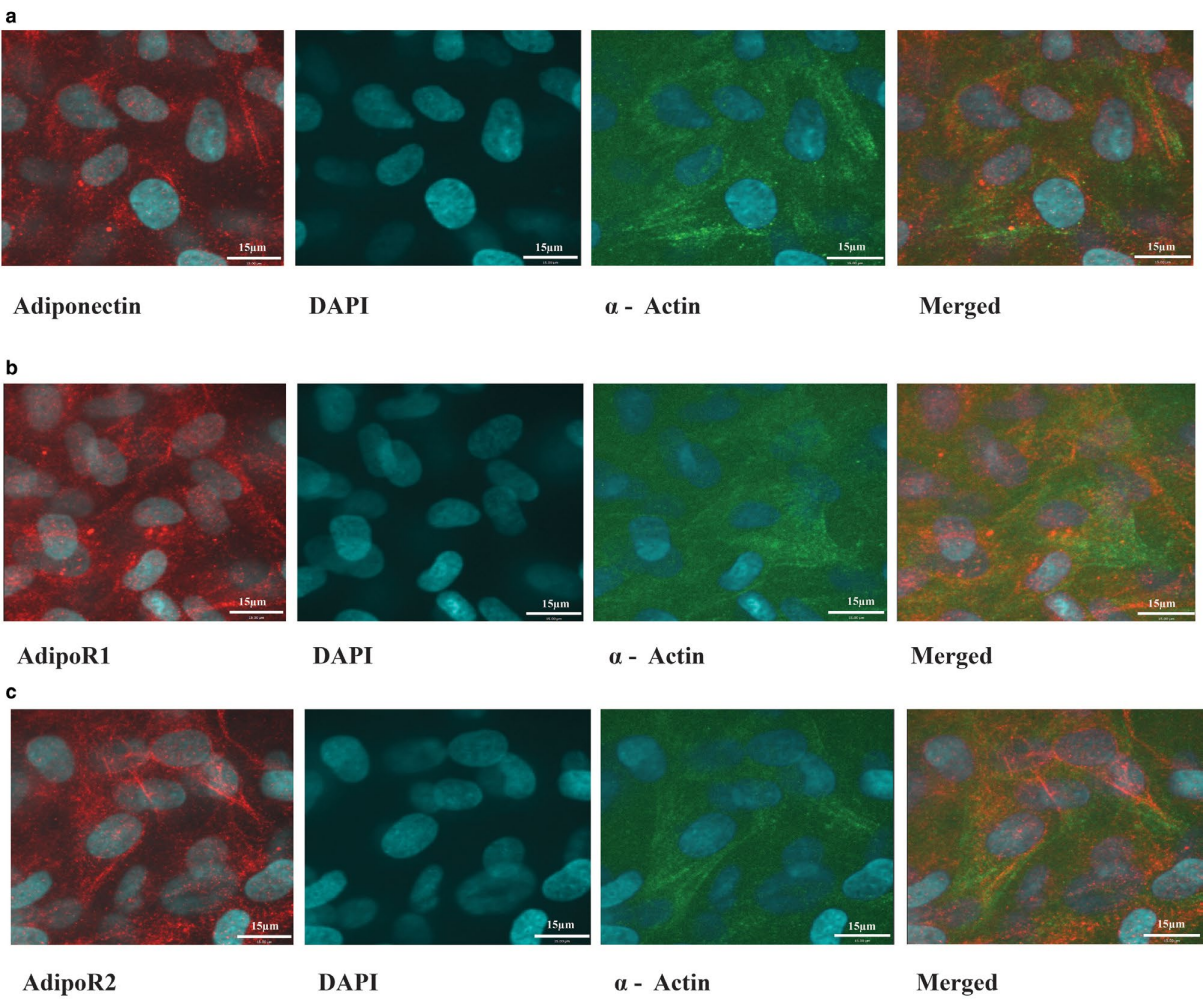
Representative photomicrographs of immunocytochemistry showing the co‐expression of APN, AdipoR1, or AdipoR2 with α‐actin in PASMC, in vitro. APN (a), AdipoR1 (b), and AdipoR2 (c) are depicted as red in the cytoplasm; α‐actin, a marker for smooth muscle, is depicted as green. Nuclear localization was visualized by DAPI, blue nuclei. Merged photomicrographs show co‐localized expression (yellow) of the protein of interest with α‐actin. All images were captured with confocal microscopy

**FIGURE 3:**
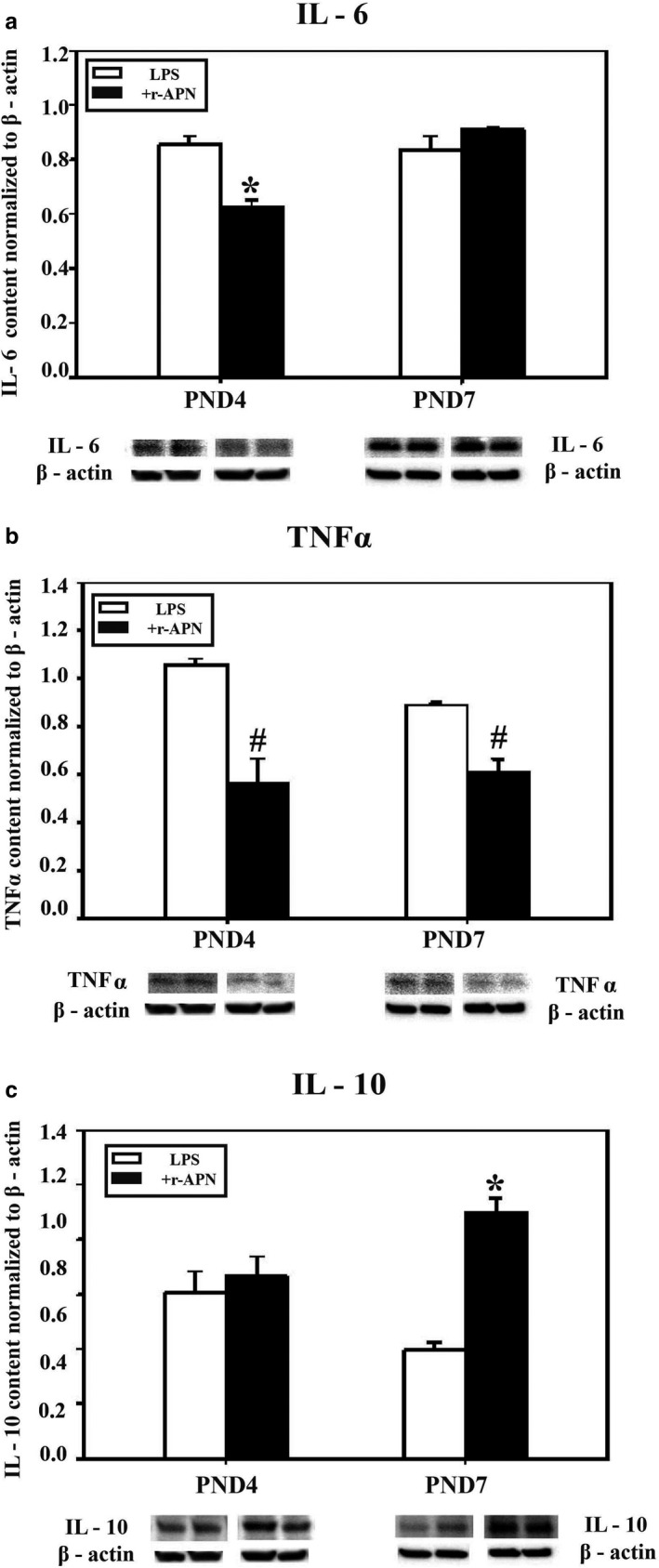
Effect of rAPN on LPS‐induced cytokine expression in PASMCs. To determine the effect of rAPN on LPS‐induced expression in PASMCs during development, PASMCs from PND4 and PND7 rat pups were treated with rAPN (20 µg/ml) or with LPS (3 µg/ml) + rAPN (20 µg/ml) (a–c) in culture for 48 hr (*n* = 4 per group); afterward the cells were homogenized and protein extracted from western blot (WB). Immunoblots were normalized to β‐actin. Bars represent mean ± *SEM*.*p < .05 by one‐way ANOVA followed by post hoc analyses using Holm–Sidak test compared to all other groups; #*p* < .05 compared to corresponding control groups. This experiment was repeated four times

Inflammation during early lung development induces PASMC proliferation, vascular remodeling, and pulmonary hypertension (Mourani & Abman, 2015). After establishing that AdipoR1, AdipoR2, and APN were co‐localized with α‐smooth muscle actin, a marker for PASMCs (Figure 2a–c), we investigated the effect of rAPN (20 µg/ml) on LPS‐induced cytokine protein expression in PASMCs at two ages, in vitro. In LPS (3 µg/ml)‐treated PASMCs from PND4 rats, rAPN decreased the expression of IL‐6 (Figure 3a) and TNF‐α (Figure 3b) (*p* < .05 vs. LPS‐treated cells) without significantly changing IL‐10 levels (Figure 3c). At PND7, rAPN had a different effect on LPS‐induced cytokine expression in PASMCs—rAPN decreased TNF‐α (Figure 3b) and increased IL‐10 (Figure 3c; *p* < .05 vs. LPS‐treated cells), while IL‐6 levels did not change (Figure 3a).

### 3.3 The protective effect of exogenous rAPN on LPS‐induced inflammation in the lung of PND4 animals, in vivo

**FIGURE 4:**
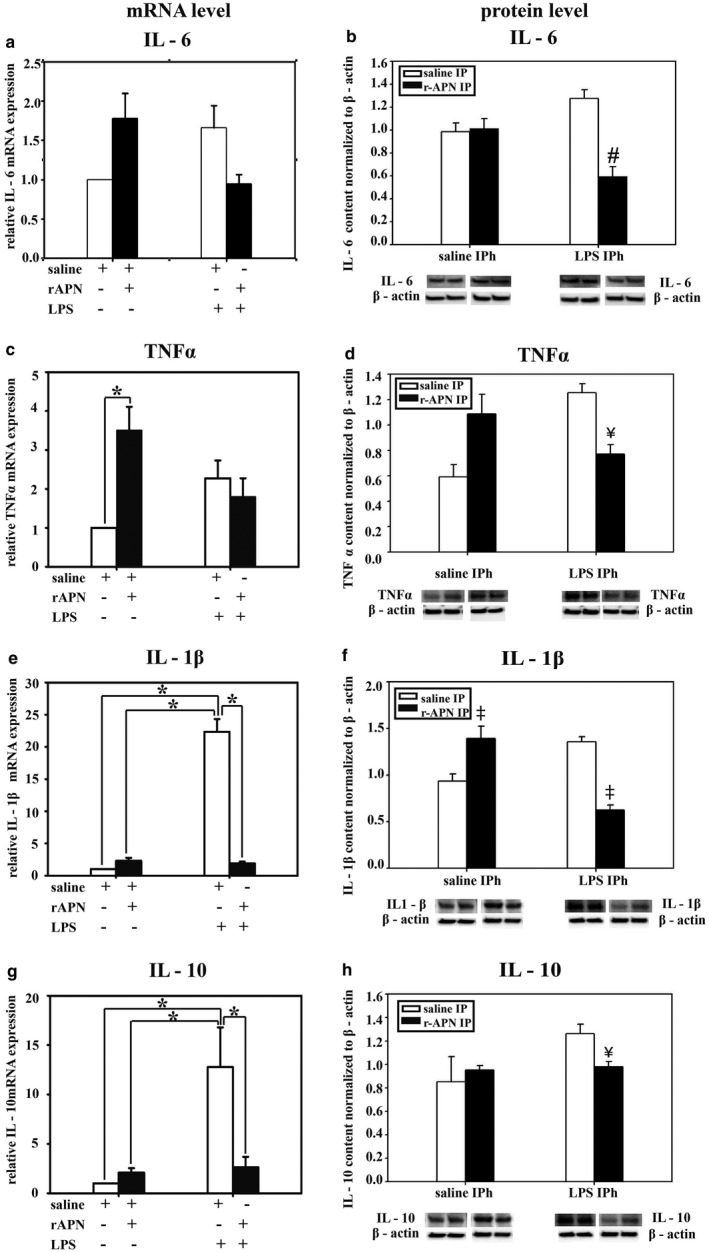
Effect of rAPN on the expression of lung mRNA and protein levels for inflammatory cytokines in response to aspiration of LPS, in vivo. PND4 rat pups were treated with rAPN (2 mg/kg) or saline by IP injections and, after 4 hr, LPS (5 mg/kg) or saline was administered to the posterior pharynx (intrapharyngeal, IPh). After 24 hr, mRNA (left panel) or protein levels (right panel) were measured in lung homogenates for IL‐6 (a and b), TNF‐α (c and d), IL‐1β (e and f), and IL‐10 (g and h) (*n* = 4 per group). Change in relative mRNA expression was calculated by 2ΔCt method normalized to *β‐actin* mRNA expression. Immunoblots were normalized to β‐actin protein expression. Bars represent mean ± *SEM*.*p < .05 by one‐way ANOVA followed by post hoc analyses using Holm–Sidak test compared to certain other groups. # represents *p* < .05 compared to all other groups. ¥ represents *p* < .05 compared to saline + LPS group. ‡ represents *p* < .05 compared to saline and saline + LPS groups

To explore whether rAPN could protect the lung from LPS‐induced injury in vivo, we investigated the effect of rAPN (2 mg/kg) given 4 hr before or 20 hr after IPh LPS (5 mg/kg) on cytokine levels and histopathology in the lungs of PND4 rats. Preliminary experiments confirmed that animals aspirated the LPS that was administered in the posterior pharynx, as evidenced by increased mRNA and protein expression of inflammatory cytokines in lung homogenates 24 hr after LPS exposure (data not shown). The cytokines and chemokines we chose to measure are elevated in the blood or tracheal aspirates of premature infants who develop BPD (Ambalavanan et al., 2009). mRNA and protein levels are shown in Figure 4a, c, e, g and Figure 4b, d, f, h, respectively. In animals pretreated with rAPN, LPS‐induced mRNA expression was reduced by 11‐fold for *IL‐1β* and by fivefold for *IL‐10* when compared to animals pretreated with saline (Figure 4e and g; *p* < .0001, vs. saline/LPS). Moreover, in animals pretreated with rAPN the expression of IL‐6, TNF‐α, and IL‐1β protein was 2.6‐, 1.7‐, and 2.6‐fold less, respectively, than in saline‐treated control animals (Figure 4b, d and f; *p* < .05 vs. saline/LPS), while protein expression for IL‐10 was minimally reduced (Figure 4h). Although animals who received rAPN in the absence of LPS had greater protein expression of *TNF‐α*, mRNA, and IL‐1β than that of control animals (Figure 4c and f; *p* < .05 vs. saline), in the presence of LPS, pretreatment with rAPN decreased LPS‐induced TNF‐α protein and *IL‐1β* mRNA and protein expression (Figure 4c and d; *p* < .05 vs. saline/LPS).

**FIGURE 5:**
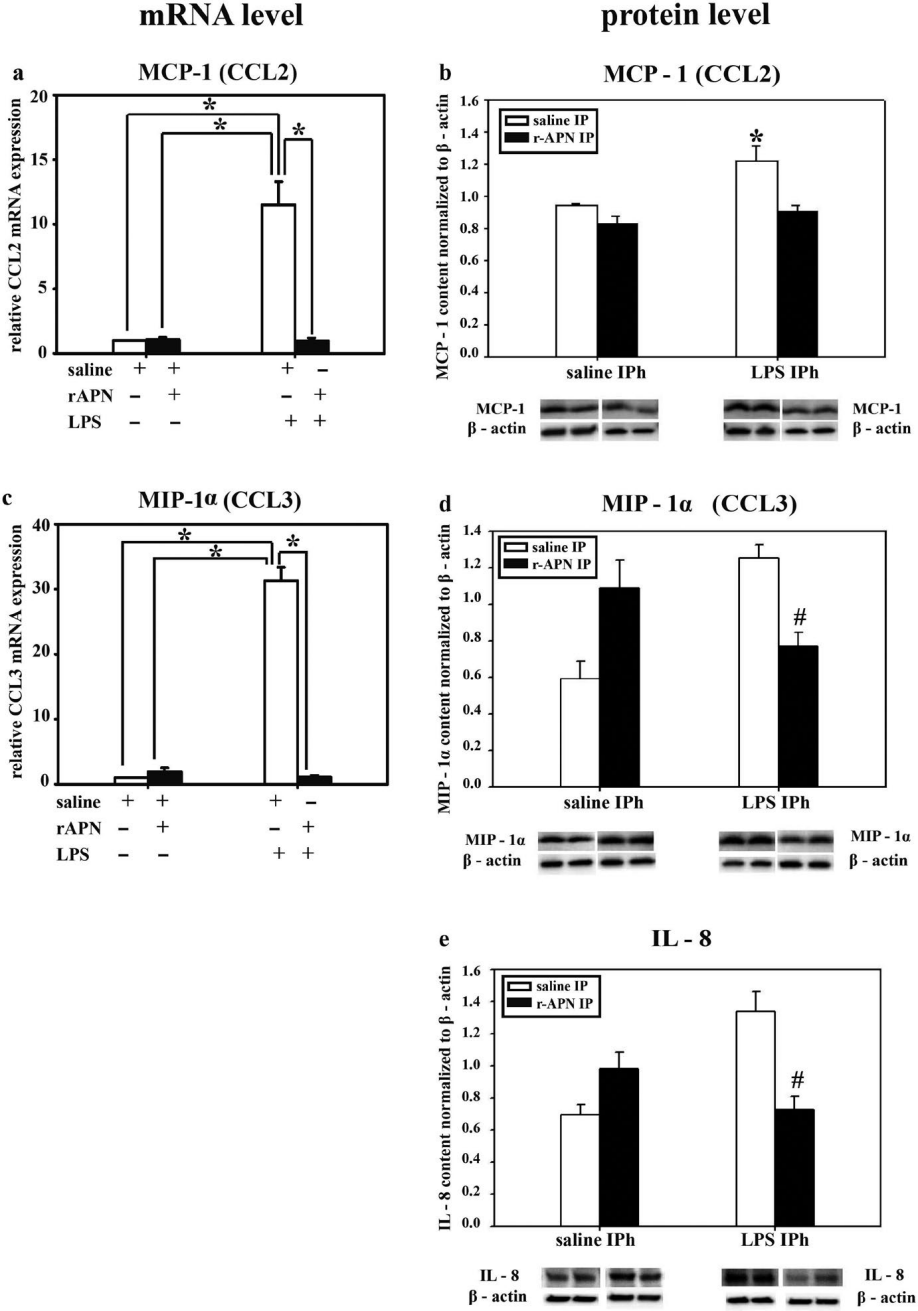
Effect of rAPN on the expression of lung mRNA and protein levels for inflammatory chemokines in response to aspiration of LPS, in vivo. PND4 pups were treated with rAPN (2 mg/kg) or saline by IP injections, and after 4 hr, LPS (5 mg/kg) or saline were administered by IPh route. After 24 hr, lungs were collected and processed for qPCR and western blot (WB). mRNA relative expression of *MCP‐1* and *MIP‐1α* (a, c); and protein content of MCP‐1, MIP‐1α, and IL‐8 (b, d, e) was measured (*n* = 4 per group). mRNA relative expression change was calculated by 2ΔCt method normalized to *β‐actin* mRNA expression. Immunoblots were normalized to β‐actin and representative immunoblots under each graph show two contiguous lanes for each group. Bars represent mean ± *SEM* by one‐way ANOVA followed by post hoc analyses using Holm–Sidak test. * represents *p* < .05 compared to all other groups and # represents *p* < .05 compared to saline IP + LPS IPh group

MCP‐1, MIP‐1α, and IL‐8, potent chemokines for migration and activation of monocytes/macrophages, are elevated in premature infants with lung injury (Allen & Kurdowska, 2014; Baier, Loggins, & Kruger, 2002). In animals pretreated with rAPN, the LPS‐induced relative mRNA expression for *MCP‐1* and *MIP‐1α* was reduced by 12‐ and 27‐fold, respectively (Figure 5a and c; *p* < .001 vs. saline/LPS). Moreover, at the same 24‐hr time point, lung protein expression for MCP‐1, MIP‐1α, and IL‐8 was 50% less in animals pretreated with rAPN before LPS when compared to animals pretreated with saline (Figure 5b, d and e; *p* < .05 vs. saline/LPS).

### 3.4 rAPN reduces the expression of immune cell markers in the lung induced by LPS exposure

**FIGURE 6:**
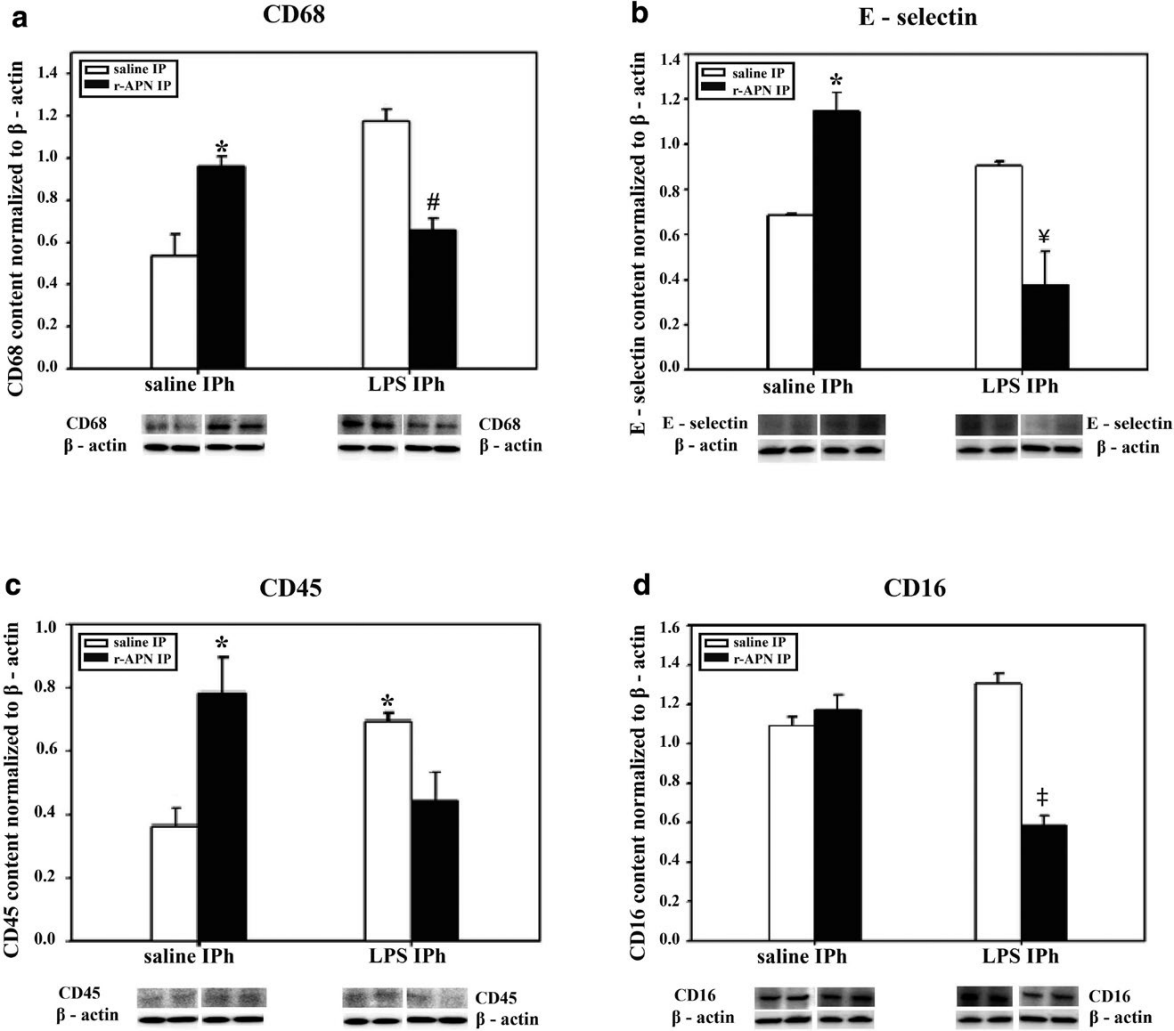
Effect of rAPN on LPS‐induced expression of inflammatory cell markers. PND4 rat pups were treated with rAPN (2 mg/kg) or saline by IP injections and, after 4 hr, LPS (5 mg/kg) or saline was administered by IPh route. After 24 hr, CD68 (a), E‐selectin (b), CD45 (c), and CD16 (d) protein levels were measured in lung homogenates by western blot (WB; *n* = 4 per group). Bars represent mean ± *SEM*. * represents *p* < .05 by one‐way ANOVA followed by post hoc analyses using Holm–Sidak test compared to saline group. # represents *p* < .05 compared to rAPN/saline group and saline/LPS group. ¥ represents *p* < .05 compared to rAPN/saline and saline/LPS. ‡ represents *p* < .05 compared to all other groups

**FIGURE 7:**
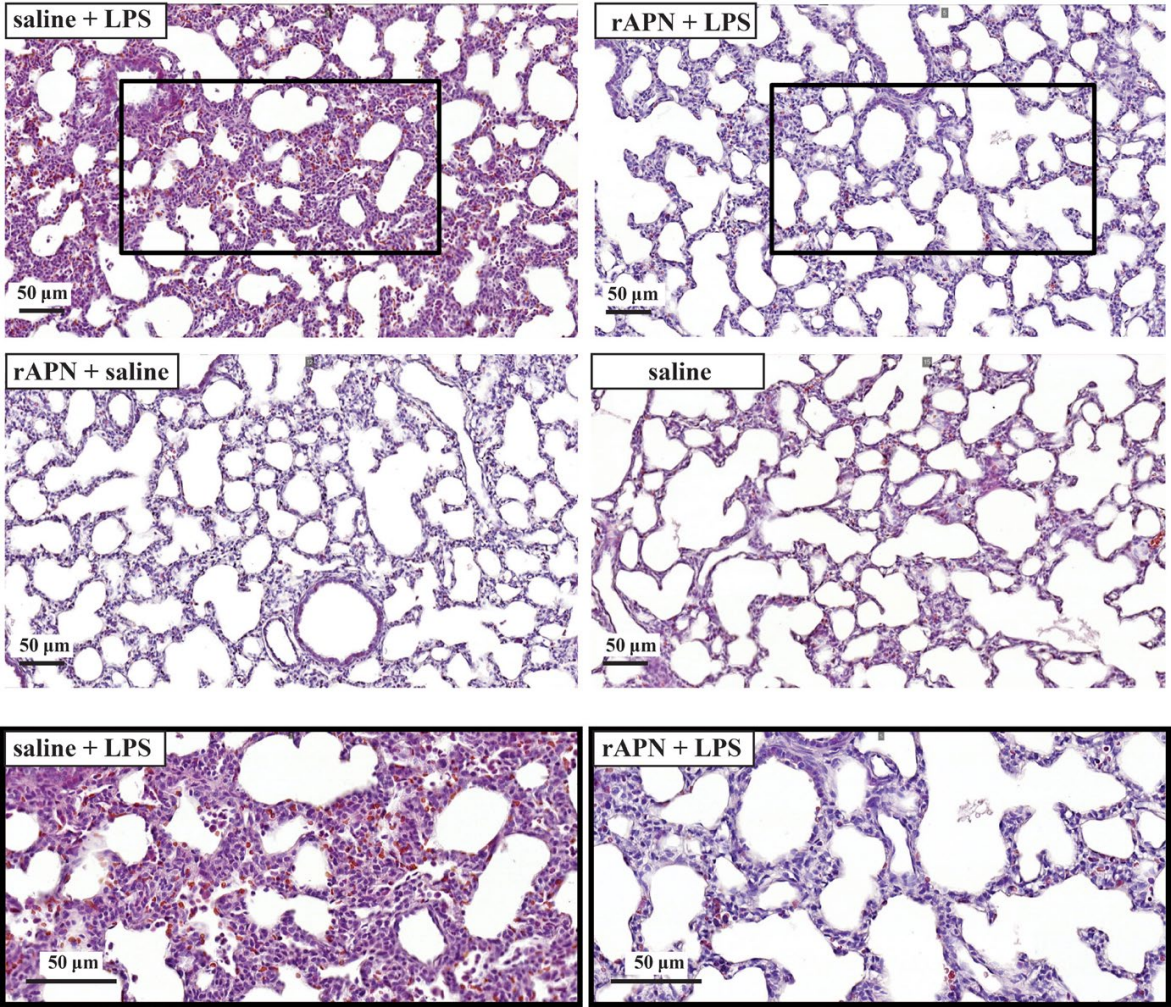
rAPN given prior to LPS decreases lung histopathology. Representative photomicrographs of lung sections from animals treated with saline/LPS (top and bottom left hand), rAPN/LPS and bottom (top right hand), rAPN/saline (middle left hand), saline/saline (middle right hand). PND4 rat pups were treated with rAPN (2 mg/kg) or saline by IP injections and, after 4 hr LPS (5 mg/kg) or saline was administered IPh. Twenty‐four hours later, animals were given an additional dose of saline or rAPN IP, and 48 hr later lungs were inflated, paraffin‐embedded, and stained with H&E (*n* = 4 per group). LPS‐exposed animals had thickened alveolar septa and cellular infiltrate in the interstitium of the lung that was not observed in the animals treated with rAPN/LPS or the other two treatment groups (rAPN/saline; saline/saline control). Bottom images are magnification of insets in top panel

**FIGURE 8:**
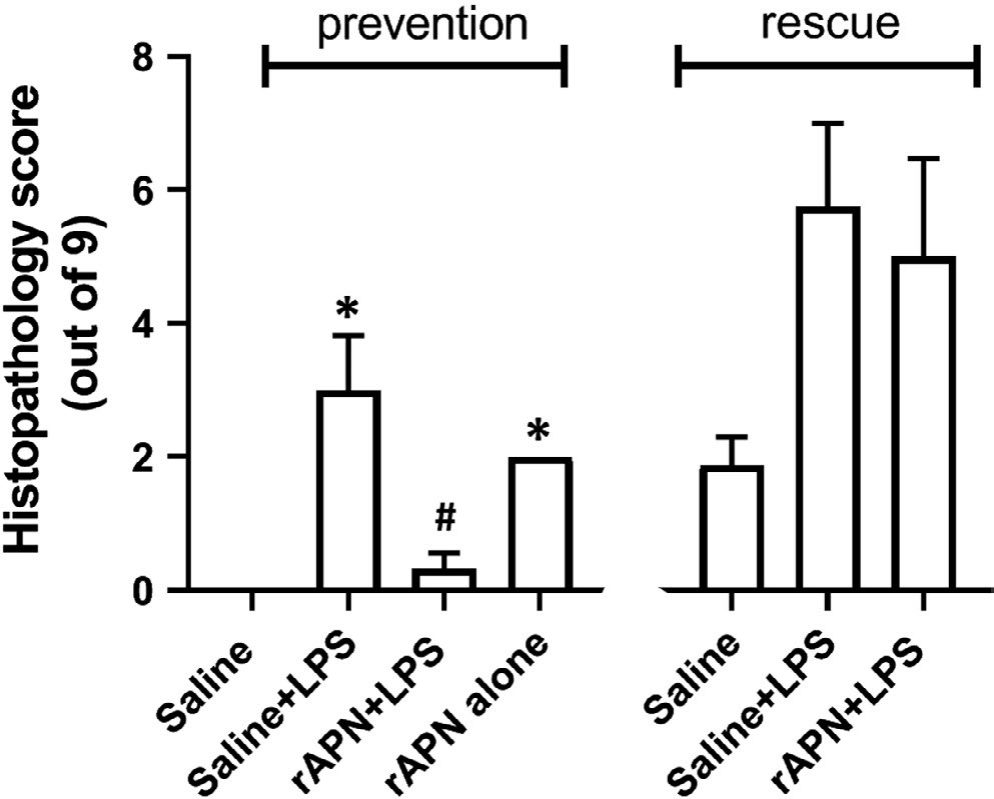
Effect of rAPN on LPS‐induced lung histopathology score. Total lung histopathology scores were determined for PND4 rats who were given saline or rAPN IP 4 hr before, and 24 hr after IPh LPS (prevention protocol) or given rAPN 20 hr and 48 after IPh LPS (rescue protocol). The lung was removed and processed for histology 72 hr after initial LPS exposure in both protocols. rAPN given before LPS reduced the histopathology score while rAPN given 20 hr after LPS did not. Bars represent mean ± *SD* one‐way ANOVA followed by post hoc analyses using Tukey's test to compare scores between groups in the prevention protocol and rescue protocol separately. * represents *p* < .02 saline versus saline + LPS and rAPN alone. ^+^ represents *p* = .003 versus saline + LPS in the prevention protocol; *N* = 4 in each group. Four treatment groups were in the prevention protocol while only three treatment groups were in the rescue protocol

Using WB to quantitate protein levels of white blood cell markers and activated endothelial cells, we found that rAPN treatment prior to LPS administration downregulated CD68, CD16, and E‐selectin protein expression by ~50% (Figure 6a, b and d; *p* < .05 vs. saline/LPS). CD45 (pan‐hematopoietic marker) protein expression was also decreased, but this difference did not reach statistical significance (Figure 6c). In an additional group of animals, 72 hr after LPS exposure, with and without rAPN pretreatment (*n* = 4 per group), histopathological changes—perivascular and interstitial red blood cell extravasation in interstitium—were evident in saline/LPS‐treated animals that were not observed in rAPN/LPS‐treated animals (Figure 7). Histopathology score differed among the treatment groups (ANOVA, *p* = .01) with rAPN/LPS‐treated animals having a lower score than saline/LPS‐treated animals (*p* = .003, Tukey's multiple comparison; Figure 8, prevention protocol).

### 3.5 The effect of exogenous rAPN on established LPS‐induced inflammation in the lung of PND4 animals, in vivo: rescue

**FIGURE 9:**
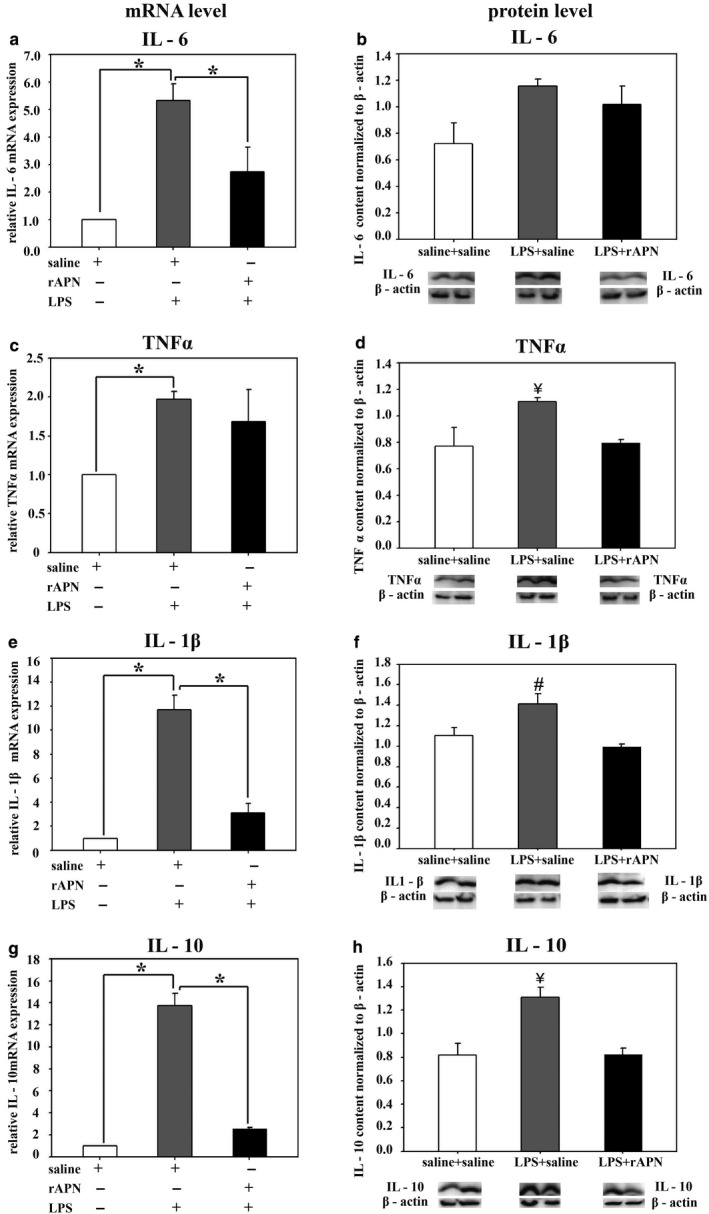
Effect of rAPN given after aspiration of LPS on mRNA and protein expression for inflammatory cytokines, in vivo, rescue. PND4 rats were given LPS (5 mg/kg) or saline IPh followed by rAPN (2 mg/kg) or saline by IP injections 20 hr after LPS. (*n* = 4 per group). Six hours after rAPN and 24 hr after LPS was given, lungs were removed and processed for mRNA (left panel) or protein (right panel) detection for IL‐6 (a and b), TNF‐α (c and d), IL‐1β (e and f), and IL‐10 (g and h). Change in relative mRNA expression was calculated by 2ΔCt method normalized to *β‐actin* mRNA expression. Immunoblots were normalized to β‐actin protein expression. Bars represent mean ± *SEM*. One‐way ANOVA followed by post hoc analyses using Holm–Sidak test *p < .05 compared to other groups; ¥ represents *p* < .05 compared to saline/saline and LPS/rAPN treatment groups for TNF‐α and IL‐10; # represents *p* < .05 compared to saline/saline and LPS/rAPN treatment groups for IL‐1β

**FIGURE 10:**
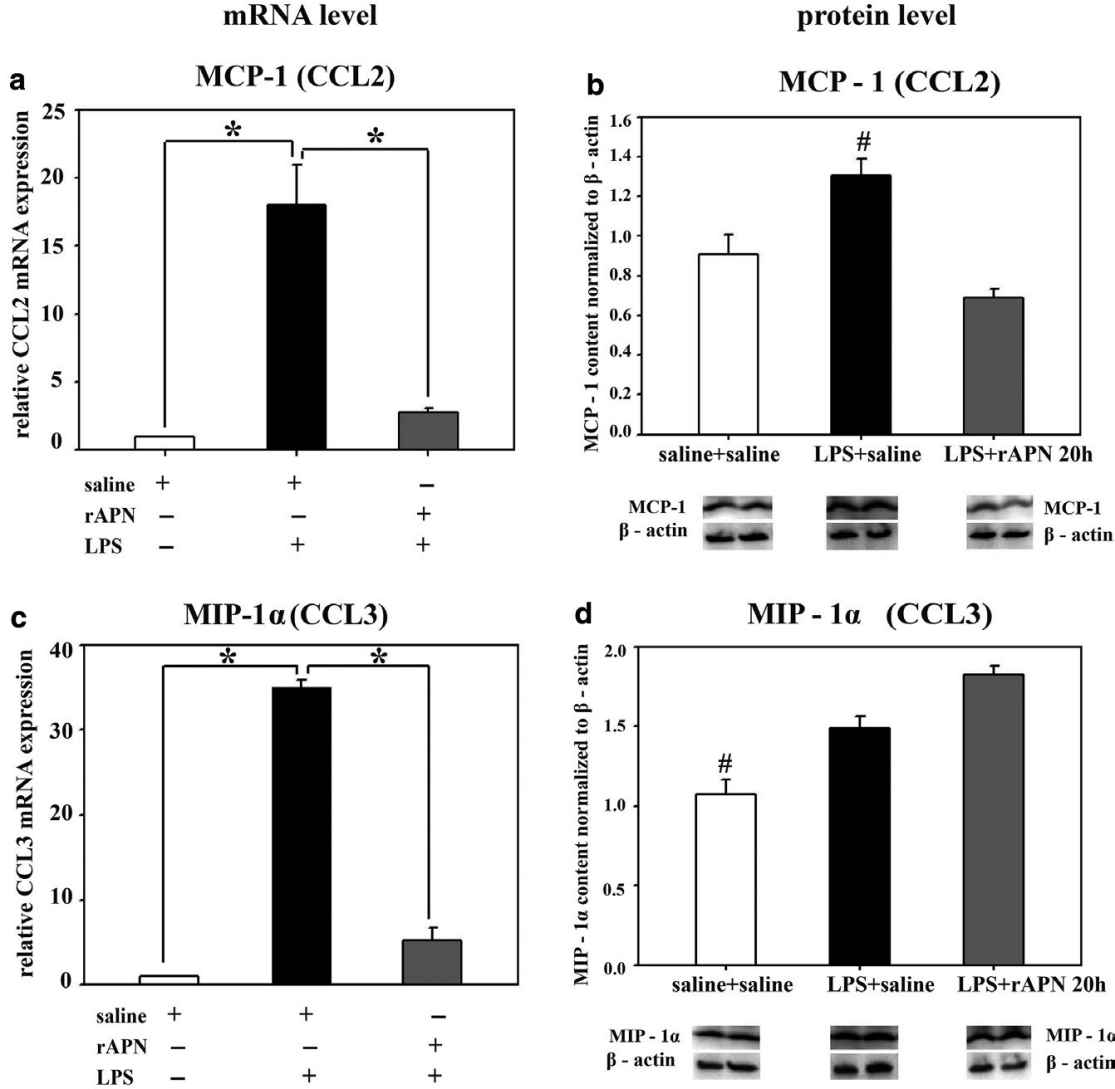
Effect of the rAPN when given as rescue treatment on LPS‐induced lung chemokine production, in vivo. PND4 rats were given LPS (5 mg/kg) or saline IPh followed by rAPN (2 mg/kg) or saline by IP injections 20 hr after LPS (*n* = 4 per group). Six hours after rAPN was given, lungs were removed and processed for mRNA (left panel) or protein (right panel) detection of MCP‐1 (a and b) and MIP‐1α (c and d). Change in relative mRNA expression was calculated by 2ΔCt method normalized to *β‐actin* mRNA expression. Immunoblots were normalized to β‐actin protein expression. Bars represent mean ± *SEM*. One‐way ANOVA followed by post hoc analyses using Holm–Sidak test. * represents *p* < .05 compared to other groups

In an exploratory experiment, we also determined the effect of rAPN on cytokine and chemokine expression given 20 hr after the animal had aspirated LPS. Tissue homogenates were analyzed for inflammatory markers 6 hr after the IP dose of rAPN but 26 hr after LPS. Similar to the effect of rAPN exposure on preventing lung inflammation, in animals that received rAPN 20 hr after LPS exposure, mRNA expression for *IL‐6, IL‐1β,* and *IL‐10* was 1.9‐, 3.7‐, and 5‐fold less, respectively, than in those animals that were treated with saline (Figure 9a, e and g; *p* < .0001, vs. LPS/saline); *TNF‐α* mRNA expression did not differ between the treatment groups (Figure 9c). Protein expression for TNF‐α, IL‐1β, and IL‐10 was also lower at this time point (Figure 9d, f and h; *p* < .05 vs. LPS/saline). IL‐6 protein expression was similar to that of control animals (Figure 9b). rAPN treatment 20 hr after LPS exposure also reduced chemokine expression. In animals treated with rAPN 20 hr after LPS exposure, *MCP‐1* and *MIP‐1α*, mRNA expression was 6.5‐ and 6.7‐fold less, respectively, than in animals treated with saline (Figure 10a and c; *p* < .001 vs. LPS/saline). At this time point, rAPN/LPS‐treated animals had twofold less protein expression for MCP‐1 than LPS/saline‐treated animals (Figure 10b; *p* < .05 vs. LPS/saline); MIP‐1α protein expression did not differ between these groups (Figure 10d). Although LPS‐treated animals had a higher lung histolopathology score than saline control animals, rAPN treatment 20 hr after LPS did not significantly decrease the score in contrast to its effect when given prior to LPS (Figure 8, rescue protocol). Data from this preliminary experiment support a role for rAPN in attenuating the already‐established LPS‐induced pro‐inflammatory response in the lung during the late saccular stage of development.

## 4 DISCUSSION

Prenatal and postnatal inflammation/infection during the saccular and canalicular stage of lung development in extremely premature infants substantially increases the risk of developing acute and chronic lung disease. Inflammation and hyperoxic exposure in newborn rats during similar stages of lung development can also induce acute and chronic changes in the lung similar to what occurs in the ELBW infants (Bhandari, 2014). Thus, we used an acute inflammatory model of lung injury to better define the role of APN in modulating acute lung inflammation in newborn rats during the saccular stage of development. For the first time, we report the expression profile of APN and its receptors (AdipoR1 and AdipoR2) in specific cells in the lungs of newborn rats and show in an in vivo model the protective effects of rAPN against LPS‐induced lung inflammation. Building on our previous data showing that mRNA and protein expression for APN and its receptors change from fetal day 19 to postnatal day 21 (Kang et al., 2018), we show that APN and its receptors (AdipoR1 and AdipoR2) are expressed in pulmonary fibroblasts, pulmonary epithelial cells, and PASMCs. In vitro, rAPN modified LPS‐induced cytokine expression in PASMCs obtained from PND4 and PND7 rats. In vivo, whether rAPN was given prior to LPS or 20 hr after LPS, rAPN was effective in blocking or reducing biochemical evidence of lung inflammation, and reducing histopathological changes when given prior to LPS in PND4 rats.

At PND4, rat pups complete the saccular stage of lung development; between PND4 and PND7, alveolar density increases by threefold (Massaro, Teich, Maxwell, Massaro, & Whitney, 1985; Meyrick & Reid, 1982). While protein expression for APN, AdipoR1, and AdipoR2 in whole lung homogenates does not differ between PND4 and PND7 (Kang et al., 2018), we report here the differences in levels of these proteins in lung fibroblasts, epithelial cells, and PASMCs at these ages.

Lung fibroblasts produce collagen, a major component of the extracellular matrix that is required for normal lung development (Kendall & Feghali‐Bostwick, 2014). However, lung fibroblasts can also be overactivated by pro‐inflammatory cytokines, reactive oxygen species, and transforming growth factor‐1β (TGF‐β1) (Toews, 2001). TGF‐β1 is a potent stimulator of fibroblast proliferation and collagen production and is required for pulmonary fibrosis (Mizikova & Morty, 2015). In animal models of BPD, TGF‐β1 levels and collagen production are increased, collagen fibers are thickened, and lungs are stiff and noncompliant, similar to findings in infants with BPD (Kotecha, Wangoo, Silverman, & Shaw, 1996; Thibeault, Mabry, Ekekezie, & Truog, 2000; Thibeault, Mabry, Ekekezie, Zhang, & Truog, 2003). AdipoR1/R2 is expressed on lung fibroblasts and rAPN via binding to AdipoR1 blocked TGF‐β and lung fibrosis (Yao et al., 2015). We show here that lung fibroblasts from rats at PND4 and PND7 express the mRNA and protein for APN and its receptors and that the expression is developmentally regulated with the expression of AdipoR1 greater at PND7, a time of more rapid alveolarization. Expression of many collagen isoforms also peaks at PND7 in newborn mice (Mizikova & Morty, 2015). Taken together, AdipoR1 signaling in lung fibroblast may modify the formation of lung fibrosis in models of injury.

APN and AdipoR1 and AdipoR2 are also expressed on lung epithelial cells (airway and alveolar) from humans and animals (Miller, Cho, Pham, Ramsdell, & Broide, 2009; Nigro et al., 2013). While APN’s anti‐inflammatory role in lung fibroblasts is well reported (Yao et al., 2015), in lung epithelial cells its role in mediating the inflammatory response is less clear. In unstimulated alveolar epithelial cells (A549), rAPN induces apoptosis (Nigro et al., 2013). However, in A549 cells treated with TNF‐α or IL‐1ß, rAPN improved cell viability, decreased apoptosis, inhibited NF‐ᴋβ activation, and increased release of the anti‐inflammatory cytokine IL‐10 without the change of pro‐inflammatory cytokines (Nigro et al., 2013). These data suggest that endogenous APN may exert differential effects depending on the microenvironment. While elevated levels of APN may have anti‐inflammatory effects during inflammation, APN may induce inflammation during normal homeostasis. Our data also show that exogenous APN in a noninflammatory state increased inflammatory cytokine mRNA and protein expression in the lungs of rats but did not produce histopathological changes.

PASMCs produce and secrete inflammatory mediators (Wang, Han, Mo, Huang, & Wang, 2017; Weng et al., 2011). Toll‐like receptor‐4 (TLR4) on PASMCs induces IFN‐γ and NF‐κB, stimulating further TNF‐α and IL‐1 secretion (Cohen, 2014); moreover, LPS binds directly to TLR4 (Lu, Yeh, & Ohashi, 2008). Our in vitro data show that rAPN reduced LPS‐induced pro‐inflammatory cytokine expression in cells from both PND4 and PND7 animals but increased the expression of IL‐10, an anti‐inflammatory cytokine, only in cells from PND7 animals. Protein expression for both AdipoR1 and AdipoR2 receptors increased between PND4 compared to PND7. These data suggest that AdipoR1/R2 receptor signaling regulating IL‐10 expression may differ during development.

Our in vivo study shows that LPS‐induced cytokine and chemokine production may be modified by exogenous APN. In addition to cytokines, LPS‐induced TLR4 receptor stimulation also stimulates the production of chemokines that attract neutrophils and macrophages to the site of injury. We studied IL‐8, MCP‐1, and MIP‐1α, because they are elevated in the tracheal aspirates of infants during the first few days of life who then develop BPD (Munshi, Niu, Siddiq, & Parton, 1997; Niu, Munshi, Siddiq, & Parton, 1998). IL‐8 is a neutrophil chemoattractant produced by epithelial cells early in the inflammatory cascade that plays a pivotal role in recruitment of inflammatory cells into the lung (Allen & Kurdowska, 2014). Monocytes, fibroblasts, vascular endothelial cells, and vascular smooth muscle cells produce MCP‐1 and its transcription is regulated by NF‐κB, a master transcription factor for inflammatory cytokines (Liu, Zhang, Joo, & Sun, 2017). Binding of APN to its receptors blocks the production of NF‐κB (Ouchi et al., 2000). We hypothesized that the increase in pro‐inflammatory cytokines (IL‐6, IL‐1β, TNF‐α) and chemokine mRNA and protein expression after IPh LPS exposure would be blocked by pre‐ or posttreatment with IP rAPN. However, there were two findings we did not expect: rAPN in the absence of LPS induced TNF‐α mRNA and protein over basal levels, and rAPN attenuated the LPS‐induced upregulation of anti‐inflammatory cytokine IL‐10, in vivo. While APN blocks the expression of TNF‐α in adipocytes (Cawthorn & Sethi, 2008), rAPN increased *TNF‐α* mRNA expression four‐ to five‐fold and increased *TNF‐α* promoter activity 60‐fold over baseline in RAW264.7 macrophages, in vitro (Park et al., 2008). A similar mechanism of TNF‐α regulation has been described for other resident tissue macrophages, such as Kupffer cells in the liver (Huang, Park, McMullen, & Nagy, 2008). It is not known whether upon initial exposure APN upregulates TNF‐α expression in other cells. We found that rAPN decreased LPS‐induced TNF‐α levels in PASMCs in vitro in both age groups, and in PND4 animals in vivo.

In addition to LPS inducing pro‐inflammatory cytokines, it also induces the expression of anti‐inflammatory cytokines, specifically IL‐10 (Chanteux, Guisset, Pilette, & Sibille, 2007). We were, however, surprised to see that pretreatment with rAPN blocked the LPS‐upregulation of *IL‐10* mRNA expression—but not protein levels—in lung homogenates to a similar degree as the effect of rAPN on transcription of mRNA for the potent pro‐inflammatory cytokine *IL‐1β*. Of note, deficiency in IL‐10, is associated with more rapid resolution of some infection and clearance of intracellular pathogens (Iyer & Cheng, 2012). IL‐10 can be a biomarker for poor disease outcome; higher blood levels of IL‐10 was predictive of BPD/death in a cohort of premature infants (Ambalavanan et al., 2009).Thus, downregulation of IL‐10 by rAPN could be of potential benefit.

There are several limitations to this study. We did not differentiate the airway from the alveolar epithelial cells when exploring APN and AdipoR1/R2 expression in lung epithelial cells. We also did not study the effect of rAPN on lung endothelial cells. Recent evidence implicates APN as key in protecting the lung endothelium from LPS‐induced lung injury in adult animals (Shah, Torres, & Bhandari, 2019). Since interstitial edema is often seen early in premature infants that progress to BPD, determining how APN interacts with lung endothelium in health and disease during development would be of interest. Another potential limitation is that a dose response was not done for LPS and rAPN. Transgenic mice with APN levels three times greater than wild‐type mice are protected from hyperoxic lung injury (Sliman et al., 2013). Thus, in preliminary experiments using newborn rats, we determined the dose and time to achieve plasma APN levels two to three times greater than baseline levels after IP injection. An additional dose of rAPN was given 24 hr after LPS exposure, and while this dose was effective in blocking the inflammatory cascade and lung injury, rAPN in the absence of LPS also increased the expression of some pro‐inflammatory cytokines. We do not know the minimal effective dose of rAPN to maximize its anti‐inflammatory effect after LPS exposure and to minimize its pro‐inflammatory effects when there is no inflammation at baseline. The dose of LPS was chosen based on the work of others (Konter et al., 2012; McGrath‐Morrow et al., 2015).

Lastly, to minimize the number of animals we elected to assay for mRNA expression and protein levels in lung homogenates at the same time, 24 hr after LPS exposure. For several of the cytokines, we found that mRNA and protein levels were not always consistent. In general, mRNA levels do not exactly predict protein levels, which can be attributed to the delay between transcription and translation and to posttranscriptional processes that determine mRNA stability (Liu, Beyer, & Aebersold, 2016). Moreover, gene and protein expression profiles for each of the cytokines and chemokines are known to differ as described by Kraus et al. in oral epithelial cells (Kraus et al., 2012). For example, in newborn infants, in response to systemic infection TNF‐α levels rise and fall prior to the changes in other pro‐inflammatory cytokines (Khaertynov et al., 2017). We decided that protein expression was more important to measure, thus we chose to assay for changes at the 24 hr time point after exposure. Even at this time point, we observed changes in gene expression for many of the cytokines in our model of injury.

**FIGURE 11:**
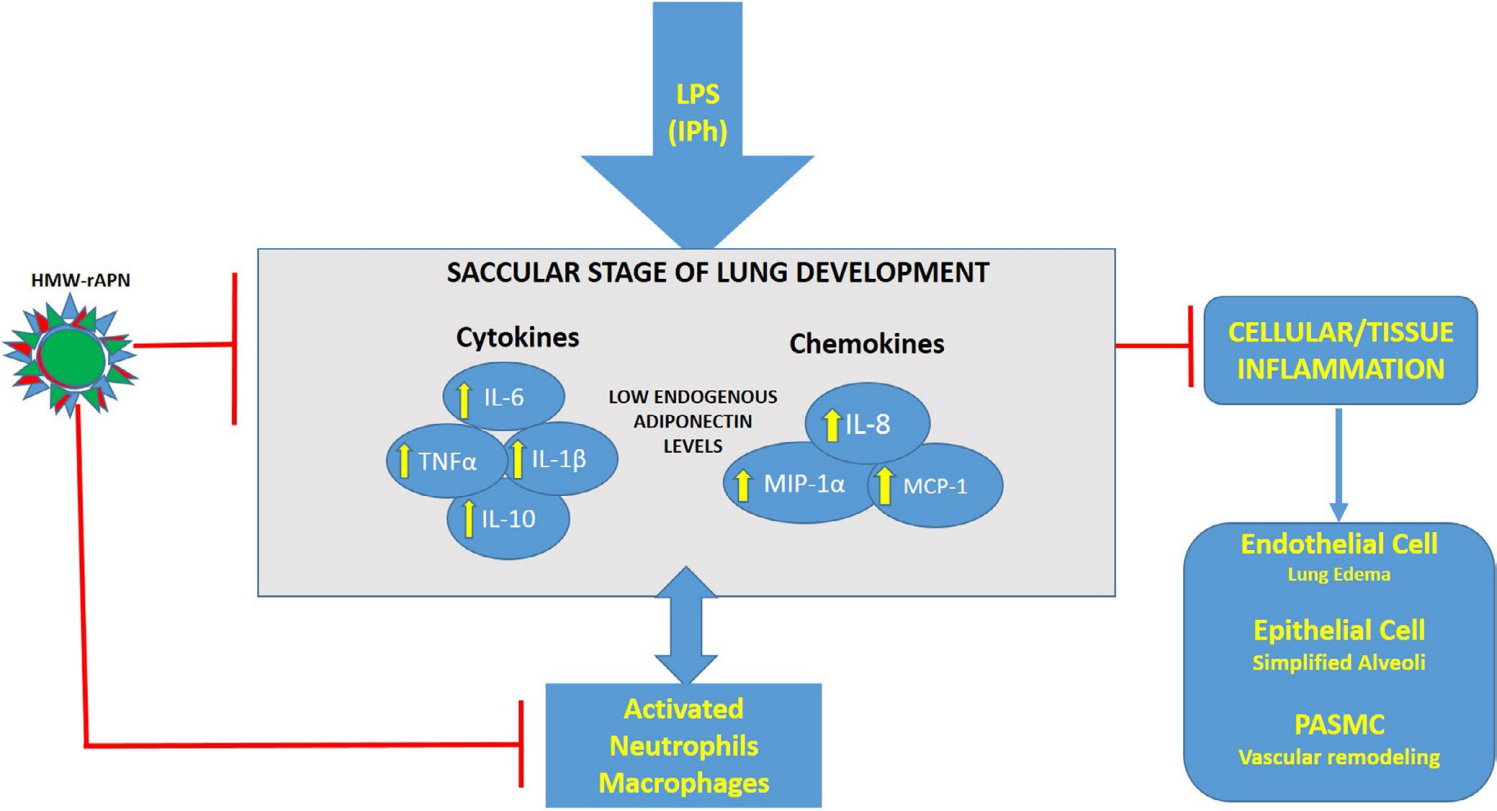
Schematic diagram summarizing the proposed downstream effects of rAPN on LPS‐induced lung inflammation during the saccular stage of lung development. rAPN blocks upregulation of inflammatory cytokines and chemokines, activation of neutrophils, macrophages, endothelial, epithelial, pulmonary artery smooth muscle cells in the lung, ultimately blocking endothelial leak and alveolar damage, and vascular remodeling. HMW—rAPN is high molecular weight—recombinant adiponectin; PASMC is pulmonary artery smooth muscle cells.

In summary, we believe our findings are clinically relevant; however, they should be interpreted cautiously due to the differences in rat versus human lung development. Rat lungs lack bronchi and adjacent pulmonary arteries compared to human lungs (Townsley, 2012); but of importance and relevance to our model, newborn rats at term are born in the saccular stage of lung development (O'Reilly & Thebaud, 2014) similar to premature infants at risk for acute and chronic lung injury from inflammation. As depicted in the schematic (Figure 11), our data support the model that LPS, by binding to TLR4 receptors on macrophages, neutrophils, and other resident lung cells, increases the production of pro‐inflammatory cytokines and chemokines leading to increased inflitration of more inflammatory cells lung parenchyma. We show that rAPN blocks the deleterious pro‐inflammatory effects of LPS on the immature lung whether it is given prior to or after the onset of inflammation.

These novel findings are relevant to what occurs in premature infants whose lungs are affected by prenatal exposure to inflammation, those infants who have ventilation‐associated pneumonia, and those who develop acute respiratory distress syndrome (ARDS) from the “cytokine storm” that occurs in infants who develop necrotizing enterocolitis and bacteremia. These data support the need for additional preclinical and clinical studies to determine whether increasing endogenous APN levels or using small molecules that can bind to AdipoR1 and AdipoR2 receptor agonists may abrogate inflammatory injury during early lung development that contributes to BPD.

## CONFLICT OF INTEREST

The authors have no conflict of interest to declare.

## AUTHORS CONTRIBUTIONS

Julijana Ivanovska, MSc: Senior research manager who performed or was involved in all experiments and supervised the trainees in the laboratory. She analyzed the data, created the figures, and wrote the first draft of the manuscript. Na‐Young Cindy Kang: Medical student at the University of Toronto who worked for several summers in the laboratory and participated in early experiments describing the expression profile using western blot for adiponectin and adiponectin receptors in the lung. She assisted with writing and editing the manuscript. Nikola Ivanovski: Undergraduate student majoring in biomedical sciences. He contributed substantially to the rescue experiments, performing qPCR, western blot, histology staining, and data analysis. Nikola also assisted Ms. Ivanovska in treating the animals and processing the tissues. Avita Nagy, MD, FRCPath, Pathologist who designed the histopathological score and scored the slides but was unaware of the treatment groups. She reviewed the final version of the manuscript. Jaques Belik, MD, Senior research scientist who contributed to the design of the experiments, interpretation of the data, and drafting and editing of the final versions of the manuscript. Estelle B. Gauda, M.D.: Principal investigator who proposed the hypothesis, helped to design experiments to test the hypothesis, reviewed all data (primary and composite), and wrote a substantial portion of the final manuscript and is the corresponding author.
